# Opening of Cx43-formed hemichannels mediates the Ca^2+^ signaling associated with endothelial cell migration

**DOI:** 10.1186/s13062-023-00408-3

**Published:** 2023-08-28

**Authors:** Hilda Espinoza, Xavier F. Figueroa

**Affiliations:** 1 Departamento de Fisiología, Facultad de Ciencias Biológicas, Pontificia Universidad Católica de Chile, Santiago, 8330025 Chile; 2 Escuela de Medicina, Facultad de Ciencias de la Salud, Universidad del Alba, Santiago, 8370007 Chile

**Keywords:** Endothelial cells, Migration, Angiogenesis, Ca^2+^ signaling, Connexin hemichannels

## Abstract

Endothelial cell migration is a key process in angiogenesis. Progress of endothelial cell migration is orchestrated by coordinated generation of Ca^2+^ signals through a mechanism organized in caveolar microdomains. Connexins (Cx) play a central role in coordination of endothelial cell function, directly by cell-to-cell communication via gap junction and, indirectly, by the release of autocrine/paracrine signals through Cx-formed hemichannels. However, Cx hemichannels are also permeable to Ca^2+^ and Cx43 can be associated with caveolin-1, a structural protein of caveolae. We proposed that endothelial cell migration relies on Cx43 hemichannel opening. Here we show a novel mechanism of Ca^2+^ signaling in endothelial cell migration. The Ca^2+^ signaling that mediates endothelial cell migration and the subsequent tubular structure formation depended on Cx43 hemichannel opening and is associated with the translocation of Cx43 with caveolae to the rear part of the cells. These findings indicate that Cx43 hemichannels play a central role in endothelial cell migration and provide new therapeutic targets for the control of deregulated angiogenesis in pathological conditions such as cancer.

**Supplementary Information:**

The online version contains supplementary material available at 10.1186/s13062-023-00408-3.

## Introduction

Tissue functional integrity and viability relies on the accurate delivery of oxygen and nutrients through the blood flow, according to the cellular activity along the time. Although regulation of blood flow distribution through dynamic control of vasomotor tone of resistance arteries in the microcirculation can timely match the changes in cellular metabolic demand, the vessel density and the microvascular network architecture must be coherent with the physiological function of the tissue. In this context, long-term regulation of tissue irrigation depends on the growth of new blood vessels from pre-existing ones through a process known as angiogenesis [1, 2], which is stimulated by tissue requirements of oxygen as observed in different physiological and pathological conditions such as wound healing, tissue regeneration, embryonic development, and tumor growth [3].

Angiogenesis is a complex process that involves a cascade of events initiated by coordinated endothelial cell migration [4] in response to an increase in intracellular Ca^2+^ concentration ([Ca^2+^]_i_). Ca^2+^ has been recognized as a key signaling mechanism in angiogenesis [5, 6] and although Ca^2+^ release from intracellular stores plays an important role in endothelial cell migration, the prevalence of the response depends on Ca^2+^ entry from the extracellular compartment. However, the mechanisms involved in this process are controversial and have not been clearly determined [7–9]. In addition, it has been proposed that cell migration also depends on the intercellular coordination of Ca^2+^ signals and, consistent with this, blockade of gap junctions results in a reduction in both migration and tubular structure formation by endothelial cells [10, 11].

Gap junctions are intercellular channels that directly connect the cytoplasm of adjacent cells, allowing the exchange of current, ions and small signaling molecules (< 1.4 nm of diameter), such as second messengers. These intercellular channels are made up by serial docking of two hemichannels, each one provided by each neighboring cell and, in turn, hemichannels are formed by the assembly of six connexin (Cx) proteins [12]. It should be noted, however, that, in addition to form gap junctions, individual hemichannels can also be functional [13, 14]. Thus, the opening of these channels contributes to the transmembrane communication of the intra- and extracellular compartments [15, 16], which may be relevant in the control of endothelial cell function, since Cx43 hemichannels have been reported to be activated by nitric oxide (NO) through direct S-nitrosylation of this Cx protein [17, 18]. NO is synthetized by the enzyme NO synthase and, of the three isoforms of the enzyme, the endothelium express the endothelial isoform (endothelial NO synthase, eNOS). As eNOS is a Ca^2+^-dependent enzyme, the initial IP_3_-mediated Ca^2+^ signaling observed in endothelial cell migration may lead to NO production and the subsequent Cx43 hemichannel activation, which, in turn, may provide a pathway for the Ca^2+^ influx involved in the sustained component of the response. However, the possible participation of Cx43 hemichannels in endothelial cell migration remains to be determined.

In this work, we evaluated the participation of Cx43 hemichannel activation in the changes of [Ca^2+^]_i_ observed in primary cultures of mesenteric endothelial cells during endothelial cell migration and tubular-like structure formation. Our findings indicate that activation of Cx43 hemichannels by a NO-mediated signaling pathway, probably S-nitrosylation, plays a central role in the Ca^2+^-dependent mechanism of endothelial cell migration that leads to new vessel formation in angiogenesis. Furthermore, the intracellular Ca^2+^ signature of the response is consistent with the subcellular re-location to the rear part of the cells of Cx43 in parallel with caveolae.

## Results

**Fig. 1:**
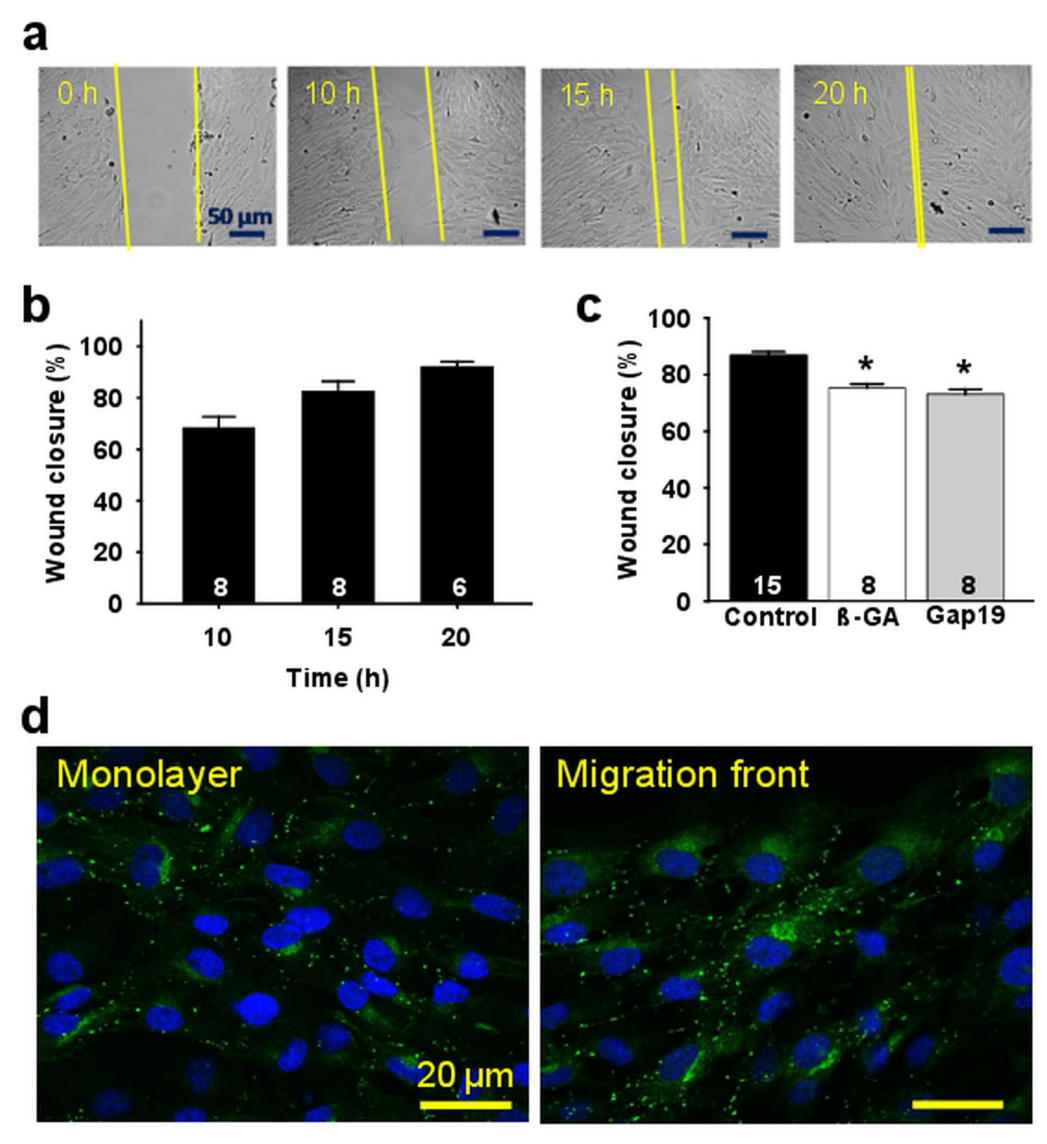
Cx43 hemichannels are involved in endothelial cell migration. **a**, Representative images of the endothelial cell migration observed in the wound-healing assay in control conditions. Primary cultures of mesenteric endothelial cells were prepared for wound-healing assay and cell migration was analyzed at 0, 10, 15 or 20 h after scratching the monolayer. Yellow lines are only intended to highlight the migration front and are not a reference for migration analysis. **b**, Quantitative analysis of the endothelial cells movement in control conditions into the cell-free scratched area at different time points (10, 15 and 20 h). **c**, Quantitative analysis of the endothelial cell migration observed before (Control) and after the treatment with 50 µM 18-ß-Glycyrrhetenic acid (ß-GA), a general Cx-formed channel inhibitor, or 300 µM TAT-Gap19 (Gap19), a specific Cx43 hemichannel blocking peptide. **d**, Representative images of immunofluorescence analysis of Cx43 expression in primary cultures of mesenteric endothelial cells in control conditions (monolayer) and in the migration front 4 h after scratching the monolayer. Cell nuclei are highlighted by the staining with DAPI (blue). Numbers inside the bars indicate the n value. Values are means ± SEM. *, P < 0.05 vs. Control by one-way ANOVA plus Bonferroni post hoc test

The two-dimension migration rate of mesenteric endothelial cells was evaluated along the time through the wound-healing assay. Migration was monitored at different time points and, in control conditions, endothelial cells showed a continuous movement into the cell-free scratched area, reaching an apparent complete wound closure after ~ 20 h (Fig. 1a and b). Therefore, the time point of 15 h was selected to analyze the participation of Cx-formed channels in endothelial cell migration, since this intermediate time point allows assessing an inhibitory or a potentiating effect on the response.

**Fig. 2:**
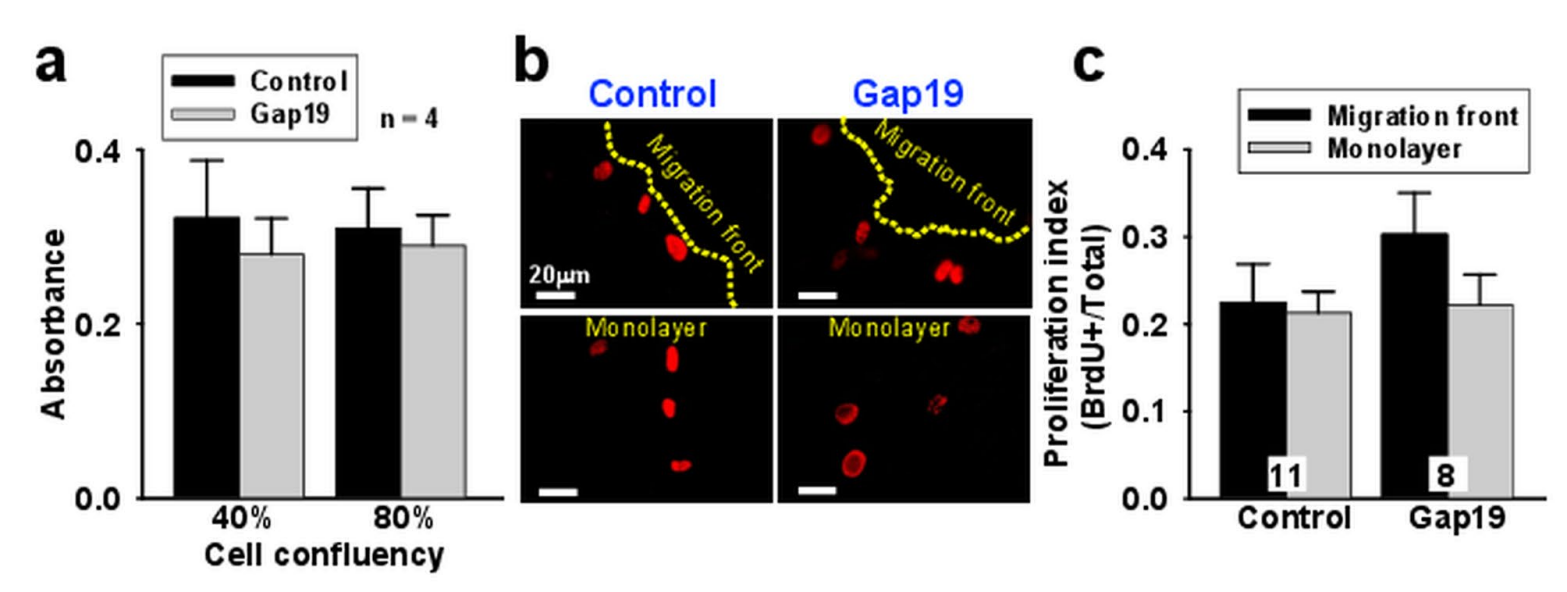
Cx43 hemichannels are not involved in endothelial cell proliferation. **a**, Analysis of endothelial cell proliferation by the bromodeoxyuridine (BrdU) incorporation assay in control conditions and after the treatment with the Cx43 hemichannel blocking peptide TAT-Gap19 (Gap19, 300 µM). Cell proliferation was evaluated in primary cultures of mesenteric endothelial cells at 40% or at 80% of confluence. **b**, Representative images of the immunofluorescence detection of BrdU incorporation into endothelial cells of the migration front and the monolayer in control conditions or in the presence of Gap19. **c**, Fluorescence intensity analysis of the experiments shown in **b**. BrdU+ denotes endothelial cells positive for BrdU. Numbers inside the bars indicate the n value. Values are means ± SEM

To evaluate the involvement of Cxs, cultures of mesenteric endothelial cells were treated with 50 µM 18-ß-Glycyrrhetenic acid (ß-GA), a general Cx-formed channel inhibitor, or with 300 µM TAT-Gap19, a specific Cx43 hemichannel blocking peptide, which evoked a clear reduction in endothelial cell migration (Fig. 1c). Interestingly, the magnitude of the inhibition observed in the presence of TAT-Gap19 was similar to that attained with ß-GA, supporting the participation of Cx43-formed hemichannels in the response. In line with this result, the expression of Cx43 in the monolayer and in the migration front was confirmed by immunofluorescence analysis and the presence of this Cx was not altered by the treatment with TAT-Gap19 (Fig. 1d and Supplementary Fig. 1, Additional file 1). In addition, the effect of Cx43 hemichannel blockade on the wound healing was not associated with a reduction in cell proliferation, as shown by the bromodeoxyuridine (BrdU) assay in cultures at 40% and 80% of confluency (Fig. 2a). Consistent with this, direct visualization of BrdU positive cells confirmed that the treatment with the peptide TAT-Gap19 did not affect the proliferation of either endothelial cells located at the migration front or at the monolayer (Fig. 2c and d), which indicate that the reduction observed in the closure of the scratched area after blocking Cx43 hemichannels only reflects the inhibition of endothelial cell migration, without the interference of a change in cell proliferation.

### Cx43 hemichannels contribute to the Ca^2+^ signaling associated with endothelial cell migration

**Fig. 3:**
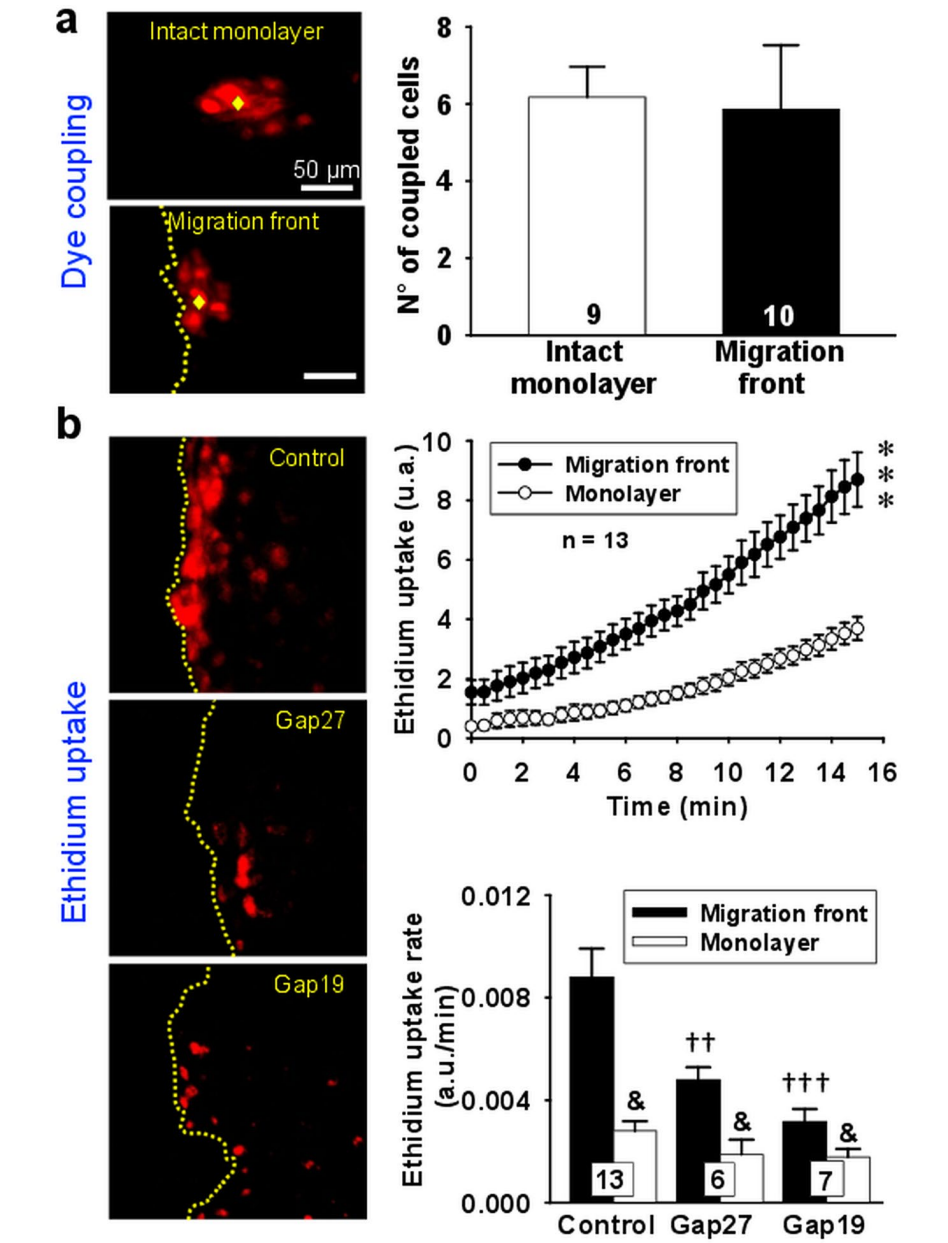
Cx43-formed hemichannels, but not gap junction channels, are associated with endothelial cell migration. **a**, Representative images of dye coupling assay (left) and the analysis of the number of coupled cells via gap junction communication (right) attained in the intact monolayer and in the migration front after scratching the monolayer. Dye coupling was assessed by measuring after 2 min the diffusion to neighboring cells (coupled cells) of the ethidium bromide microinjected into a single endothelial cell. The yellow diamond indicates the microinjected cell. **b**, Representative images of the ethidium uptake observed in primary cultures of mesenteric endothelial cells in control conditions and in the presence of the Cx blocking peptide ^37,43^Gap27 (200 µM) or the Cx43 hemichannel inhibitor TAT-Gap19 (Gap19, 300 µM) (left). Ethidium uptake was evaluated 15 min after scratching the monolayer and cells were incubated with the dye for 15 min, as shown in the time course of ethidium uptake observed in the intact monolayer and in the migration front (b, right top). Dot yellow lines depict the edge of the migration front. In addition, the analysis of the ethidium uptake rate achieved in the intact monolayer and in the migration front in control conditions and in the presence of ^37,43^Gap27 or Gap19 is also shown (b, right bottom). The rate of ethidium uptake was assessed by calculating the slope of the increase in fluorescence intensity along the time. Changes in ethidium-fluorescence signal are expressed in arbitrary units (a.u.). Numbers inside the bars indicate the n value. Values are means ± SEM. ***, P < 0.001 vs. Monolayer by two-way ANOVA. ††, P < 0.01 and †††, P < 0.001 vs. Migration front in control conditions (Control) by one-way ANOVA plus Bonferroni post hoc test. &, P < 0.001 vs. Migration front by paired Student’s t-test

Endothelial cell migration depends on coordinated activation of Ca^2+^ signals and Cx-formed channels have been shown to provide a functional pathway for Ca^2+^ communication [19, 20]. Interestingly, migration of endothelial cells in the wound-healing assay was not associated with a change in the level of gap junction-mediated intercellular communication, as compared with that observed previous to scratch the intact monolayer (Fig. 3a). However, in contrast to the gap junction communication, the activity of hemichannels was dramatically increased, since a striking increment in ethidium uptake was attained in the first two rows of endothelial cells from the migration front 15 min after scratching the monolayer (Fig. 3b). The increase in ethidium uptake was abolished by the treatment for 10 min with the Cx43 blocking peptide ^37,43^Gap27 or the Cx43 hemichannels specific inhibitor TAT-Gap19 (Fig. 3b), which is consistent with the hypothesis that endothelial cell migration is associated with the opening of Cx43-formed hemichannels.

**Fig. 4:**
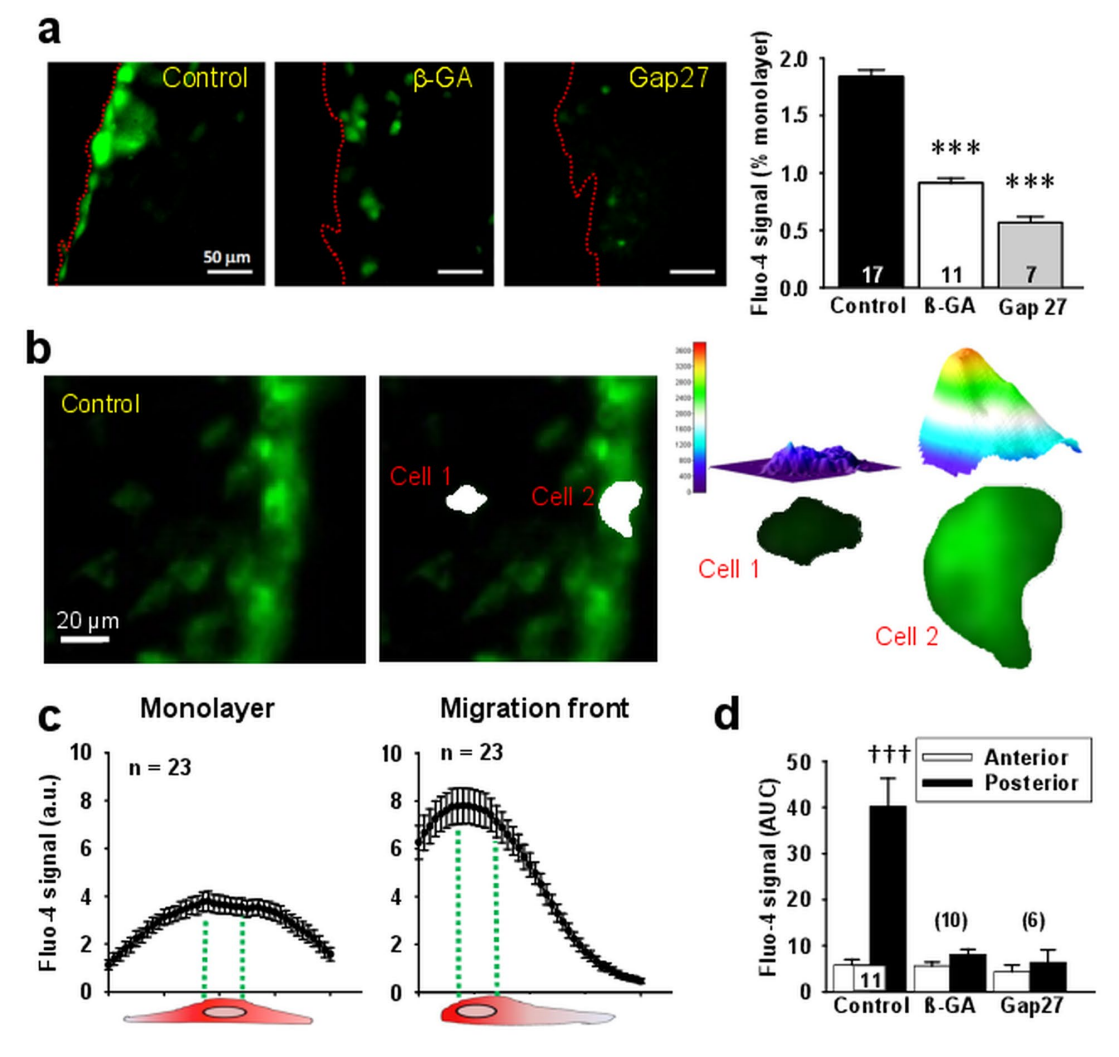
Endothelial cell migration depends on a Cx43-formed channel-mediated increase in intracellular Ca^2+^ concentration ([Ca^2+^]_i_). **a**, Representative images (left) and fluorescence intensity analysis (right) of the increase in [Ca^2+^]_i_ observed in endothelial cells of the migration front 15 min after scratching the monolayer in control conditions and in the presence of 50 µM 18-β-Glycyrrhetenic acid (ß-GA), a general Cx-formed channel blocker, or 200 µM ^37,43^Gap27, a peptide designed to block channels formed by Cx37 or Cx43. Variations in the levels of [Ca^2+^]_i_ were assessed with the fluorescent Ca^2+^ indicator Fluo-4. **b**, Representative images of the changes in [Ca^2+^]_i_ of endothelial cells (left) in which the differences in the subcellular distribution of the Ca^2+^ signal attained in a cell of the migration front (Cell 2) and a cell within the monolayer (Cell 1) are highlighted in a 3D analysis (middle and right). **c**, Fluorescence intensity analysis of the Fluo-4 signal measured along the endothelial cells length (from back to front) in the migration front and the monolayer. **d**, Analysis of the changes in [Ca^2+^]_i_ levels attained in the rear and anterior edge of endothelial cells of the migration front in control conditions and after the treatment with ß-GA or ^37,43^Gap27. Changes in Fluo-4 signal are expressed as the area under the curve (AUC). Note that cells were treated with the Cx blocking peptide ^37,43^Gap27 for only 10 min to inhibit Cx hemichannels, without affecting gap junction channels. Dot red lines depict the edge of the migration front. Numbers inside the bars or in parentheses indicate the n value. Values are means ± SEM. ***, P < 0.001 vs. Control by one-way ANOVA plus Bonferroni post hoc test. †††, P < 0.001 vs. Anterior by paired Student’s t-test

As expected, the initiation of the wound-healing assay was paralleled by a prominent increase in [Ca^2+^]_i_ in the endothelial cell migration front and Cx43 hemichannels are permeable to Ca^2+^ [3, 21]. Therefore, we evaluated the participation of these channels in the Ca^2+^ signaling involved in the migration process. In line with the opening of Cx43 hemichannels, a strong increase in [Ca^2+^]_i_ was observed exclusively in the migration front, which showed the same pattern of the increment in ethidium uptake and was blocked by ß-GA, the peptide ^37,43^Gap27 (Fig. 4a) or the Cx hemichannel inhibitor La^3+^ (See Supplementary Fig. 2, Additional file 1), supporting the participation of Cx43 hemichannel opening in the Ca^2+^ signaling. In line with the importance of Ca^2+^ signaling in endothelial cell migration, the increase in [Ca^2+^]_i_ persisted during the whole response and although it slowly decayed along the time, the [Ca^2+^]_i_ only returned to resting levels when endothelial cells of both migration edge of the wounded area reached each other (See Supplementary Figs. 3 and 4, Additional file 1). As expected, application of ^37,43^Gap27 results in a progressive reduction of the Ca^2+^ signal observed 4 h after scratching the monolayer, confirming the relevance of Cx43 hemichannels during the progress of the migration process.

**Fig. 5:**
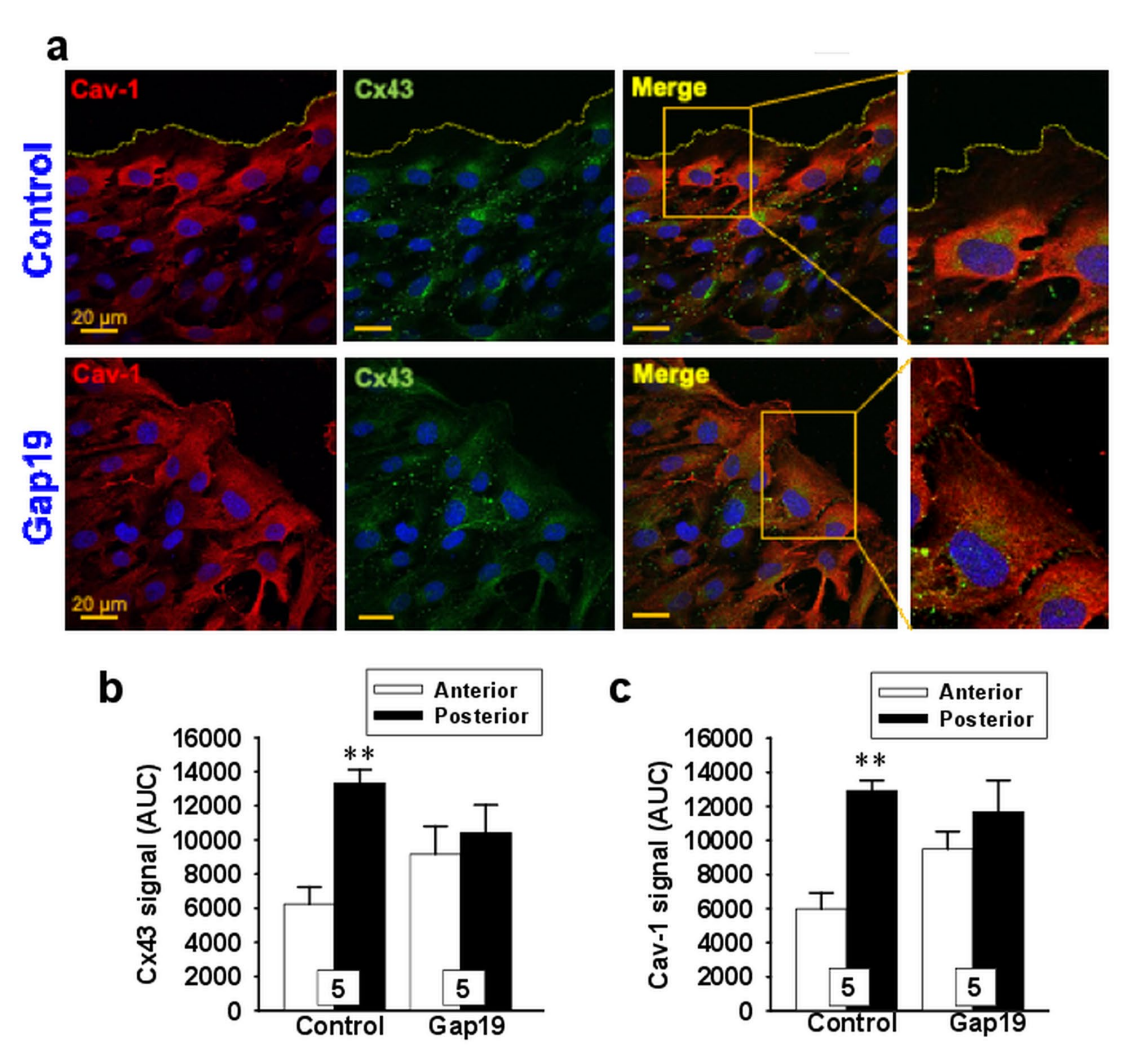
Cx43 hemichannel activation leads to subcellular re-distribution of caveolin-1 (Cav-1) and Cx43 in migrating endothelial cells. **a**, Immunofluorescence analysis of the subcellular distribution of Cav-1 (red) and Cx43 (green) in control conditions and after the treatment with 300 µM TAT-Gap19 (Gap19), a specific blocker of Cx43 hemichannels. Cell nuclei are highlighted by the staining with DAPI (blue). The square indicatres the area that was enlarged on the right. **b and c**, Densitometric analysis of Cx43 (**b**) and Cav-1 (**c**) signal in the rear and anterior part of endothelial cells of the migration front in control conditions and in the presence of Gap19. Numbers inside the bars indicate the n value. Values are means ± SEM. **, P < 0.01 vs. Anterior by paired Student’s t-test

**Fig. 6:**
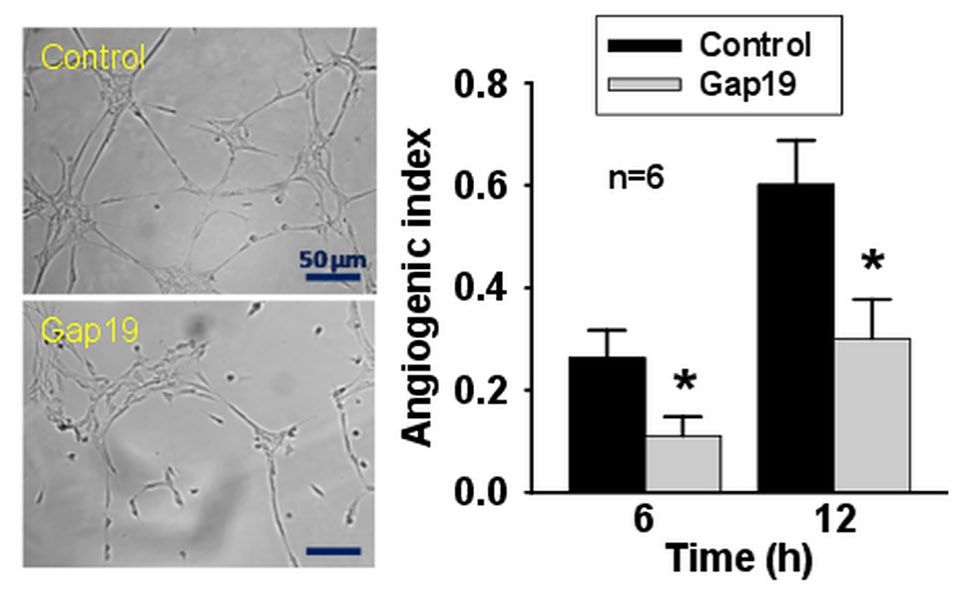
Activation of Cx43-formed hemichannels is involved in the progress of angiogenesis in vitro. Representative images of the formation of tubular-like structures in Matrigel by primary cultures of mesenteric endothelial cells after 12 h in control conditions or in the presence of 300 µM TAT-Gap19 (Gap19), a specific blocker of Cx43 hemichannels (left). In addition, the analysis of the development of tubular-like structure formation by the calculation of the angiogenic index observed after 6 and 12 h in control conditions and in the presence of Gap19 is also shown (right). Values are means ± SEM. *, P < 0.05 vs. Control by unpaired Student’s t-test

Furthermore, a more detailed analysis of this process showed that the increase in [Ca^2+^]_i_ was not homogenous throughout the cell, as this signal was markedly more prominent on the rear part of the cells and gradually decreased toward the migration edge (Fig. 4b and c), suggesting that the Ca^2+^ signaling was originated in the trailing edge of the cells. Consistent with this notion and the participation of caveolae in the polarization of the migratory response, the endothelial cell migration triggered by scratching the monolayer evoked the re-distribution of Cx43 and caveolin-1 (cav-1) to the backside of the cells (Fig. 5). It should be noted, however, that the immunofluorescence analysis did not show an apparent co-localization of both proteins in the intact monolayer or in the migration front of endothelial cells in control conditions or after the treatment with TAT-Gap19 (Fig. 5 and Supplementary Fig. 1, Additional file 1). Interestingly, ß-GA and ^37,43^Gap27 inhibited the increase in [Ca^2+^]_i_ selectively in the rear part of the cells, without affecting the Ca^2+^ level observed in the anterior side of the cells (Fig. 4d). Likewise, the treatment with TAT-Gap19 prevented the cellular re-distribution of Cx43 with cav-1 (Fig. 5) and also reduced the tubular structure formation in an in vitro angiogenesis assay (Fig. 6). Taken together these results indicate that Cx43 hemichannels provide a relevant pathway for Ca^2+^ entry during the stimulation of endothelial cell migration and the re-distribution of these channels is involved in the functional subcellular localization of the Ca^2+^ signaling.

### Cx43 hemichannel opening is mediated by NO

**Fig. 7:**
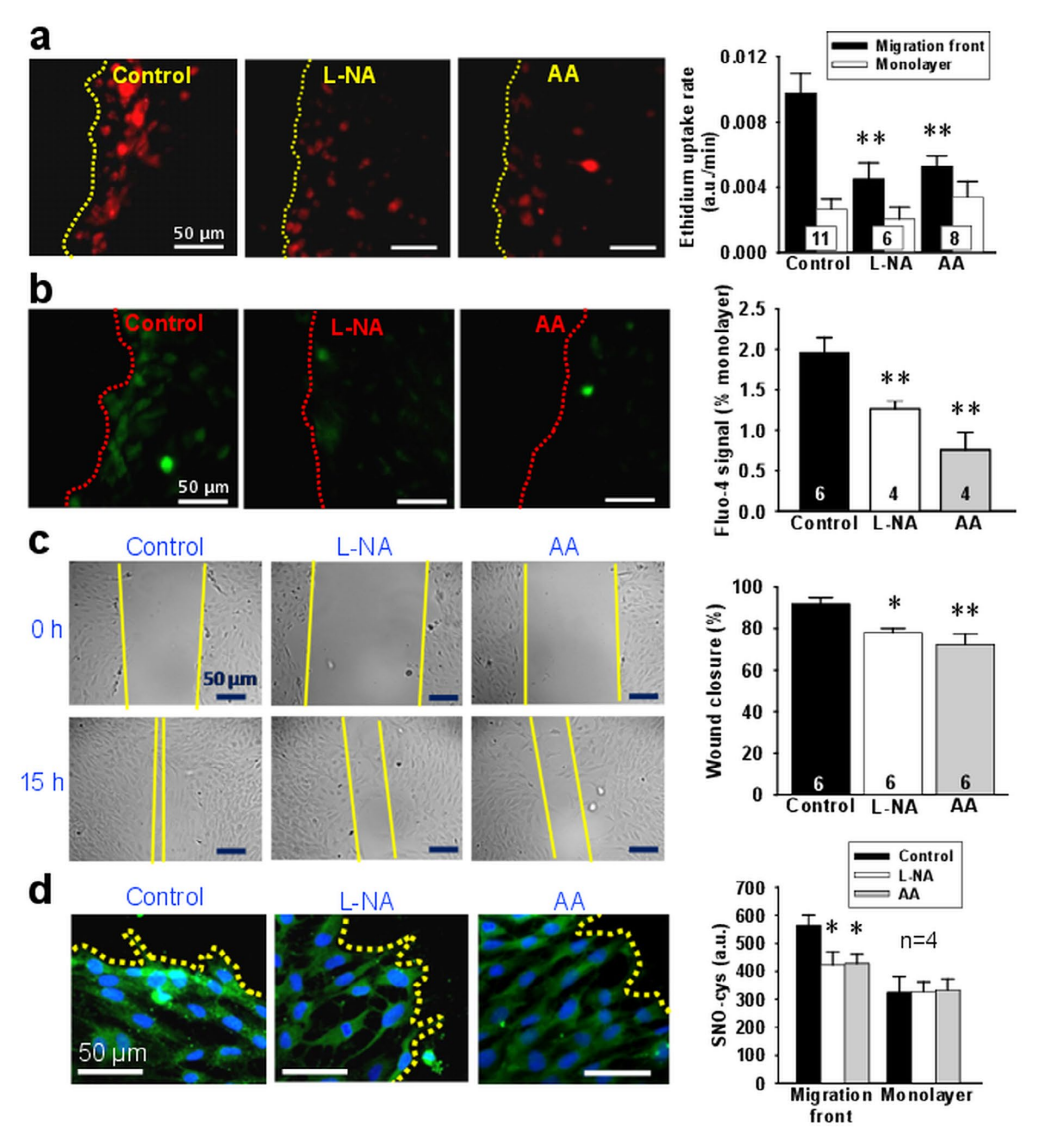
Activation of Cx43-formed hemichannels in endothelial cells of migration front depends on NO-mediated S-nitrosylation. **a**, Representative images of ethidium uptake in primary cultures of mesenteric endothelial cells (left) and the analysis of ethidium uptake rate observed in the migration front and in the monolayer in control conditions and after the treatment with 100 µM N^G^-nitro-L-arginine (L-NA) or 50 µM ascorbic acid (AA). The rate of ethidium uptake was assessed by calculating the slope of the increase in fluorescence intensity along the time. **b**, Representative images (left) and fluorescence intensity analysis (right) of the increase in [Ca^2+^]_i_ observed in endothelial cells of the migration front in control conditions and in the presence of 100 µM L-NA or 50 µM AA. Variations in the levels of [Ca^2+^]_i_ were assessed with the fluorescent Ca^2+^ indicator Fluo-4. **c**, Representative images (left) and quantitative analysis (right) of the endothelial cell migration observed in the wound-healing assay just after scratching the monolayer (0 h) and after 15 h in control conditions and in the presence of L-NA or AA. Yellow lines are only intended to highlight the migration front and are not a reference for migration analysis. **d**, Detection of total protein S-nitrosylation by immunofluorescence (left) and densitometric analysis of the fluorescence intensity (right) observed in the migration front and the monolayer of primary cultures of endothelial cells in control conditions and after the treatment with L-NA or AA. Cell nuclei are highlighted by the staining with DAPI (blue). Numbers inside the bars indicate the n value. Values are means ± SEM. *, P < 0.05 and **, P < 0.01 vs. Control by one-way ANOVA plus Bonferroni post hoc test

NO production plays a central role in the activation of endothelial cell migration and angiogenesis and, NO-mediated S-nitrosylation can trigger the opening of Cx43 hemichannels [16, 22]. Then, a NO-depended mechanism may be involved in the Cx43-mediated migration of endothelial cells. Consistent with this notion, blockade of NO production with 100 µM N^G^-nitro-L-arginine (L-NA) prevented the activation of Cx43 hemichannels and, consequently, also blocked the increase in [Ca^2+^]_i_ and inhibited the endothelial cell migration observed in the wound-healing assay (Fig. 7a and c). In addition, detection of SNO-cys radicals by immunofluorescence analysis revealed that the cells of the migration front exhibit an increase in global protein S-nitrosylation that was sensitive to the treatment with L-NA or ascorbic acid (Fig. 7d). Interestingly, the fluorescence signal for SNO-cys co-localized with the stain for Cx43 in the migration front of endothelial cell in control conditions, but not in the presence of L-NA or ascorbic acid (See Supplementary Fig. 5, Additional file 1). In line with these results, ascorbic acid also prevented the increment in ethidium uptake and [Ca^2+^]_i_ attained in the migration front as well as inhibited the endothelial cell migration (Fig. 7a and c), which supports the hypothesis that Cx43 hemichannels are activated by NO-mediated S-nitrosylation during the initiation of the endothelial cell migration.

## Discussion

Angiogenesis is a complex process that relies on fine synchronization of endothelial cell migration [1, 2]. Although migration certainly depends on the integration of several signaling pathways, Ca^2+^ signaling plays a critical role in the activation and progress of the reponse along the time [23]. Cxs have also been involved in the control of endothelial cells migration, suggesting that gap junction channels may contribute to the coordination of the Ca^2+^ signaling that commands the endothelial cell migration. However, it is not clear the mechanism by which Cxs modulate the migration process and both gap junction-dependent and -independent signaling pathways have been proposed. Interestingly, although the intercellular gap junction communication may be involved in the coordination of migrating cells, our findings indicate that the Ca^2+^ signaling supporting the initiation and progress of endothelial cell migration, and further formation of tubular structures depend on the activation of Cx43-formed hemichannels. In addition, the increase in [Ca^2+^]_i_ was associated with subcellular re-distribution of Cx43 and cav-1 to the rear part of the cells.

Gap junctions provide a critical communication pathway for coordination of endothelial cell function and, consistent with this notion, Cxs have been found to be involved in the control of endothelial cell migration and proliferation [24, 25]. Whereas proliferation is inversely correlated with the expression level of Cxs [24], endothelial cell migration has been found to be promoted by the functional presence of these proteins, specially Cx43 [10, 25, 26]. The positive effect of Cx43 on endothelial cell migration has been ascribed to gap junction-mediated communication and to gap junction-independent mechanisms [25, 27], including the regulatory spatial association of Cx43 with the tyrosine phosphatase SHP-2 [28]. Nevertheless, it is important to note that most of these studies were perfomed using macrovascular endothelial cells such as human umbilical vein endothelial cells (HUVEC), porcine aortic endothelial cells (PAEC), EA.hy926 cells or the endothelium of excised rat aorta [5], which are not located in the vascular territory that normally responds to angiogenic stimuli in physiological conditions. In line with this notion, primary cultures of mesenteric endothelial cells were used in the present study and, in agreement with previous reports, endothelial cell migration was inhibited by ß-GA, but also by the treatment with the peptide TAT-Gap19 (Fig. 1). The magnitude of the inhibition observed in the presence of ß-GA and TAT-Gap19 was similar (Fig. 1), which indicates that a signaling pathway mediated by Cx43-formed hemichannels, rather than gap junction channels, is involved in the control of endothelial cell migration. In this context, it should be noted that the inhibition of Cx43 hemichannels did not affect the endothelial cell proliferation (Fig. 2), confirming that the reduction attained in wound closure reflects exclusively the blockade of endothelial cell migration and not an effect on the number of cells at the migration front.

Consistent with the participation of Cx43-formed hemichannels in the progress of wound-induced migration observed in primary cultures of mesenteric endothelial cells, a striking increase in ethidium uptake was detected exclusively in the migration front (Fig. 3). Interestingly, the increment in ethidium uptake was restricted exclusively to the first two or three cell rows at the edge of the wounded area and, importantly, this response was almost abolished by the application of the peptides ^37,43^Gap27 or TAT-Gap19 (Fig. 3). Although the uptake of ethidium may be attributed to a potential cell damage evoked by scratching the monolayer, the sensitivity of ethidium uptake to ^37,43^Gap27 and TAT-Gap19 confirms the involvement of Cx43 hemichannels, since TAT-Gap19 blocks selectively these channels [29, 30] and ^37,43^Gap27 was applied for only 15 min. In this context, it is important to note that treatment with Cx blocking peptides for short periods of time (< 45 min) only inhibits hemichannel activity, without affecting gap junction channels, which are blocked by longer application of these peptides (> 1 h) [13, 31, 32]. In addition, although endothelial cell migration has been associated with changes in gap junction communication [10, 26, 33], we found that, in mesenteric endothelial cells, the level of intercellular coupling via gap junction was similar in the migration front as compared with that observed in endothelial cells before scratching the intact monolayer (Fig. 3). Taken together, these results confirmed the participation of Cx43 in endothelial cell migration, as previously proposed in different studies [33, 34]; however, in contrast to the involvement of a gap junction dependent or a Cx43-mediated signaling, the data strongly support that the activation of Cx43-based hemichannels play a key role in the progress of the endothelial cell migration observed in the wound-healing assay.

Hemichannels connect the intracellular compartment with the extracellular space and Cx43-formed hemichannels are permeable to Ca^2+^ [35]. Furthermore, Ca^2+^ is a critical signaling pathway for endothelial cell migration [5, 6] and the inhibition of Cx43 hemichannels activation prevented the increase in [Ca^2+^]_i_ observed after scratching the endothelial cell monolayer (Fig. 4 and Supplementary Figs. 2 and 3, Additional file 1), which suggests that these membrane channels provide an important pathway for the Ca^2+^ influx involved in the sustained phase of the response. The increment in [Ca^2+^]_i_ activated during endothelial cells migration is mediated by an initial inositol-1,4,5-trisphosphate (IP_3_)-triggered Ca^2+^ release from the endoplasmic reticulum and a subsequent sustained phase that depends on Ca^2+^ entry through a yet to be defined Ca^2+^-permeable route [5]. In this context, several mechanisms have been proposed to contribute to the Ca^2+^ influx involved in the response activated by pro-angiogenic stimuli, including store-operated Ca^2+^ entry (SOCE), STIM1 and Orai1, TRPC, Na^+^/Ca^2+^ exchanger and voltage-gated Ca^2+^ channels [5]. In addition, different intracellular Ca^2+^ signatures have been described depending on the nature and strength of the stimulation and on the vascular territory and species [5]. However, migration does not depend on a single stimulus, since it is a very complex process that requires the spatial and temporal integration of different signaling components [3, 4] and the molecular characteristics of the migration-associated Ca^2+^ signaling directly activated in response to endothelial damage has not been fully addressed. Interestingly, the long-lasting extracellular dependent-[Ca^2+^]_i_ increase observed in endothelial cells facing the injured area after damaging the rat aorta inner wall was sensitive to general blockers of Cx-formed channels [36], which is consistent with our results and highlights the importance of Cx43 hemichannels in the injury-evoked endothelial cell migration. Therefore, the data of the present work indicate that Cx43 hemichannels play a central role in the sustained phase of Ca^2+^ signaling (Fig. 4 and Supplementary Fig. 3, Additional file 1), which has been demonstrated to command the signaling pathways that determine the activation of the migration mechanism in endothelial cells [37]. In this context, it is important to note that endothelial cell migration is a key step to lead to the formation of tubular structures in the process of angiogenesis [3, 4, 38]. Then, we also evaluated the involvement of Cx43 in this response and, as expected, the formation of tubular-like structures by primary cultures of mesenteric endothelial cells was clearly reduced in the presence of TAT-Gap19 (Fig. 6), supporting the relevance of Cx43 hemichannel-mediated Ca^2+^ signaling in the formation of new vessels during angiogenesis.

Caveolae are recognized as signaling microdomains that has been shown to organize Ca^2+^ signaling [39, 40] and cav-1, a structural protein of caveolae, appears to play a central role in the control of migration of endothelial cells and other cell types [41, 42]. In migrating endothelial cells, caveolae are relocated to the rear part of the cells to coordinate the Ca^2+^ signaling that regulates the direction of the movement [43]. In line with the re-organization of the Ca^2+^ machinery during migration, Cx43 was redistributed to the rear part of the cells of the migration front in parallel with cav-1. However, although our results did not show an apparent co-localization of Cx43 with cav-1, the subcellular redistribution of these two proteins was prevented by the treatment with the Cx43 hemichannel blocker TAT-Gap19 (Fig. 5), which suggests that the response was triggered by the Ca^2+^ influx activated through the opening of Cx43 hemichannels. In addition, consistent with the re-location of cav-1 in conjuction with Cx43 and the relevance of Cx43 hemichannels in the Ca^2+^ response, the magnitude of the Ca^2+^ signal was higher in the rear part of the cells and gradually decayed toward the leading edge (Fig. 5). Therefore, these data confirm that caveolae with cav-1 play a pivotal role orchestrating the migration process and highlight the participation of Cx43 hemichannels in the control of the Ca^2+^ signaling generated in endothelial cells of the migration front.

We note that Cx-formed hemichannels are thought to be normally closed, but they have been reported to be open in response to inflammatory conditions [44, 45]. In addition, Cx43-formed hemichannels can be activated by NO-mediated S-nitrosylation [17, 18]. In endothelial cells, NO is generated by the enzyme eNOS in a Ca^2+^-dependent manner [46, 47] and as the injury-evoked migration is initiated by an IP_3_-mediated release of intracellular Ca^2+^ from the endoplasmic reticulum [36], the rise in NO production triggered by this initial Ca^2+^ signal might lead to the opening of Cx43 hemichannels through S-nitrosylation. In agreement with this hypothesis, we confirmed that the activation of Cx43 hemichannels was mediated by a NO-dependent pathway, since the ethidium uptake and the increase in [Ca^2+^]_i_ achieved in endothelial cells of the migration front, with the concomitant endothelial cell movement into the cell-free scratched area, were inhibited by L-NA (Fig. 7). Interestingly, in addition to L-NA, hemichannel opening, Ca^2+^ signaling and migration were sensitive to ascorbic acid, a reducer that can denitrosylate proteins [48]. In addition, the response was also associated with a strong increment of total protein S-nitrosylation in endothelial cells facing the edge of the wounded area (Fig. 7), which show an apparent co-localization with Cx43 (See Supplementary Fig. 5, Additional file 1). Taken together, these results support the involvement of NO-mediated S-nitrosylation in the response and indicate that activation of Cx43 hemichannels by this posttranslational modification plays a critical role in the long-lasting increase in [Ca^2+^]_i_ that directs endothelial cell migration and tubular structure formation, which may contribute to the NO-mediated mechanisms involved in the control of angiogenesis.

In summary, the results of this study support the notion that Cx43-formed hemichannels play a central role in endothelial cell migration. Stimulation of endothelial cell migration in the wound-healing assay was paralleled by the activation of Cx43 hemichannels and the subcellular redistribution of Cx43, in parallel with caveolae. In addition, our data indicate that the opening of Cx43 hemichannels, possibly by S-nitrosylation of this Cx, provides a critical pathway for Ca^2+^ entry and, consistent with the re-location of Cx43, the increase in [Ca^2+^]_i_ was higher in the rear part of endothelial cells during migration and tubular structure formation, which suggests that Cx43 hemichannels are involved in the control of angiogenesis.

## Materials and methods

### Reagents and chemicals

All chemicals of analytical grade were obtained from Merck (Darmstadt, Germany). M199 medium and fetal bovine serum (FBS) were purchased from Gibco (NY, USA). Ethidium bromide, bovine serum albumin (BSA), HEPES, MOPS, endothelial cell growth supplement (ECGS), ß-GA, L-NA were purchased from Sigma-Aldrich (MO, USA). Ascorbic acid was obtained from Santa Cruz Biotechnology (TX, USA) and the peptides ^37,43^Gap27 and TAT-Gap19 were from Tocris Bioscience (Bristol, UK).

### Animals

Male Sprague-Dawley rats (200–220 g) were bred and maintained in the Research Animal Facility of the Pontificia Universidad Católica de Chile. All studies were approved by the Institutional Bioethics Committee (protocol ID 170,823,033) and experiments were conducted according to the Helsinki Declaration. The National Institutes of Health Guide for the Care and Use of Laboratory Animals (NIH Publications No. 8523, revised 2011) were followed. All efforts were made to minimize the suffering and number of animal used.

### Primary cultures of mesenteric endothelial cells

Rats were anesthetized with xylazine and ketamine (10 and 90 mg/kg i.p., respectively) and the isolated vascular mesenteric bed was prepared as described by Figueroa et al. [49]. Briefly, the superior mesenteric artery was cannulated and perfused at 2 mL/min with sterile MOPS-buffered Tyrode solution (in mM: 118 NaCl, 5.4 KCl, 2.5 CaCl_2_, 1.2 KH_2_PO_4_, 1.2 MgSO_4_, 5 MOPS, and 11.1 glucose, anti-anti solution, pH 7.4) to wash out blood from the vessels. The aorta was cut to ensure a fast killing of the rats by exsanguination under deep anesthesia. Thus, mesenteries were excised from the intestinal wall and primary cultures of endothelial cells were prepared as described by Ashley et al. [50]. Mesenteric vessels were incubated for 1 h in sterile Tyrode solution containing 0.2% collagenase type I (Worthington, NJ, USA) and 0.1% BSA for 1 h at 37 °C, and then, the collagenase/BSA was removed by diluting the solution with cold M199 medium and centrifugation at 3000 rpm two successive times. Pellets were resuspended in complete M199 (M199 + 20% FBS + 20 µg/mL ECGS) and cells were seeded in 12 mm sterile glass coverslips located onto 24 wells plate. Three hours later, nonadherent cells were removed from the culture with PBS (in mM: 136.9 NaCl, 2.68 KCl, 10.44 NaH_2_PO_4_ and 1.76 KH_2_PO_4_, pH 7.4) and remaining cells were kept at 37 °C in a 5% CO2-95% air atmosphere at nearly 100% relative humidity. Experiments were performed using confluent cultures of endothelial cells (~ 2 days of culture) in which the culture media was replaced by a MOPS-buffered Tyrode saline solution (pH 7.4). Only one microscopy field was analyzed per coverslip and a maximum of two measurements were performed per cell culture. At least three independent cultures were evaluated per experimental group.

### Wound-healing assay

Confluent monolayer of endothelial cells in control conditions or treated for 45 min with 100 µM L-NA or for 15 min with 50 µM ß-GA, 300 µM TAT-Gap19 or 50 µM ascorbic acid were scraped using a p200 pipette tip, and then, the monolayer was gently washed with PBS to remove cell debris. The treatment with L-NA, ß-GA, TAT-Gap19 or ascorbic acid was maintained during the whole experimental period. In these experiments, the medium M199 was supplemented with only 5% FBS. Images were captured using a Nikon Eclipse E600 FN1 microscope. Changes in wounded area were evaluated using the ImageJ software and endothelial cell migration was expressed as a percentage of wound closure (%).

### BrdU incorporation assay for cell proliferation

Cell proliferation was evaluated by assessing the global BrdU incorporation or through the direct immunofluorescence analysis of BrdU incorporation. For global BrdU incorporation, the Millipore©’s BrdU Cell Proliferation Assay Kit (Merck millipore, CA, USA) was used. Endothelial cells were seeded in 96 wells plate and cell cultures with a confluence of 40 or 80% were incubated with 10 µM BrdU in control conditions or in the presence of 300 µM TAT-Gap19 for 12 h. Then, BrdU incorporated into endothelial cell nuclei was recognized using an anti-BrdU peroxidase conjugated secondary antibody. Cell proliferation was quantified by measuring absorbance of tetra-methylbenzidine (TMB) product at 450 nm. For direct detection of BrdU incorporation by immunofluorescence analysis, confluent endothelial cells seeded in 12 mm glass coverslips were scraped using a p200 pipette tip and incubated for 12 h with 10 µM BrdU (Thermo Scientific, IL, USA) in absence (Control) or presence of 300 µM TAT-Gap19. Then, cells were fixed with 4% PFA, DNA denatured using a 2 M HCl solution and blocked with 3% BSA in PBS. Coverslips were incubated overnight at 4 °C with a mouse anti-BrdU primary antibody (1:5000, Thermo Scientific, IL, USA) and then with Alexa Fluor 568 anti-mouse secondary antibody (1:1000, Molecular Probes, OR, USA) for 1 h at room temperature. Fluorescent signal was examined in endothelial cells of both the migration front and monolayer using a Olympus BX41 WI microscope and a charged-coupled device camera (ProgRes C5; Jenoptik, Jena, Germany). Endothelial cell proliferation was expressed as the proliferation index, which was calculated according to the following relation: BrdU+/Total cells, where BrdU + is the number of cells that incorporated BrdU into nuclei and Total cells is the number of cells observed in the bright field.

### Immunofluorescence analysis

Endothelial cells were fixed with 4% PFA, blocked with 3% BSA in PBS and incubated overnight at 4 °C with rabbit primary antibodies directed against cav-1 (1:100, Thermo Scientific, IL, USA), or SNO-cys (1:300, Sigma Aldrich, MO, USA) or a mouse primary antibody directed against Cx43 (1:100, BD-Transduction Labs, KY, USA), and then, with Alexa 568-labeled goat anti-rabbit or Alexa 488-labeled anti-mouse secondary antibodies (Molecular Probes, OR, USA) for 1 h at room temperature, as appropriate. The fluorescence signal was examined using a Nikon spectral C2si confocal microscope. Cx43 and cav-1 subcellular distribution in endothelial cells was analyzed along the antero-posterior axis using ImageJ software.

### Measurement of gap junction-mediated communication by dye coupling

Changes in gap junction communication were assessed by dye coupling. Endothelial cells were microinjected with a 150 mM KCl solution containing 25 mM ethidium bromide through a glass micropipette using a IM-400 microinjector (Narishige, Tokyo, Japan) and the intercellular diffusion of the dye was evaluated after 2 min. The number of coupled cells was recorded by assessing neighboring cells that accumulated the dye. Cells were considered to be coupled when the dye was transfered to two or more neighboring cells and the dye coupling was calculated as the average number of cells to which the dye spread from the microinjected cell.

### Measurement of connexin hemichannel activation

Changes in connexin hemichannel opening were evaluated by assessing ethidium uptake. Endothelial cells were incubated for 10 min with 5 µM ethidium bromide before initiating the recording of ethidium uptake by time lapse experiments. Images were acquired every 30 s for 15 min using a BX50WI microscope coupled to an intensified charged-coupled device camera (Retiga Fast 1394; QImaging, Surrey,BC, Canada) and the time course of the changes in fluorescence intensity were evaluated with the ImageJ software. In addition, the ethidium uptake rate was determined by calculating the slope of the changes in the fluorescence signal along the time.

### Measurement of changes in intracellular Ca^2+^ levels

Changes in intracellular Ca^2+^ concentration were measured using the fluorescent Ca^2+^ indicator Fluo-4 (Invitrogen, CA, USA). To upload the cells with Fluo 4, primary cultures of endothelial cells were incubated with 3 µM Fluo-4 acetoxymethyl ester (AM) for 1 h at room temperature. Fluo 4-AM was dissolved in DMSO and, thus, prepared in MOPS-buffered Tyrode saline solution. Ca^2+^ measurements were started 15 min after initiating the wound-healing assay by scraping the monolayer. The fluorescent signal was examined using an Olympus BX50 WI microscope and an intensified CCD camera (Retiga Fast 1394, QImaging). Images were acquired every 3 s for 30 s in the wounded area and monolayer. The treatment with Cx-formed channel inhibitors (50 µM ß-GA or 200 µM ^37,43^Gap27) started from 10 min before scraping the monolayer and was maintained during the whole experimental period. Changes in [Ca^2+^]_i_ were expressed as the variations of the fluorescence intensity observed in the migration front in relation to the monolayer, Ca^2 +^ _F_/Ca^2 +^ _M_, where Ca^2 +^ _F_ is the fluorescence intensity in cells of the migration front and Ca^2 +^ _M_ the fluorescence intensity in cells of the monolayer. In addition, changes in Fluo-4 signal were analyzed along the anteroposterior axis of endothelial cells and in anterior and posterior borders of cells of the migration front.

### Endothelial cell-mediated formation of tubular structures

The analysis of endothelial cells tube formation was performed in 12 mm coverslips covered with 100 µl Matrigel® (Corning, NY, USA) according to the manufacturer’s protocol. Matrigel®-solution was added to coverslips located into a 96 wells plate and allowed to solidify and polymerize at 37ºC in a 5% CO2-95% air atmosphere. Then, endothelial cells were seeded on top of the Matrigel and tubular-like structure formation was evaluated for 6 and 12 h in control conditions or in the presence of 300 µM TAT-Gap19. Seven fields per coverslip were examined using a Nikon Eclipse E600 FN1 microscope and the results were expressed as the angiogenic index according to the following formula: (Total cells + connected cells)/ total cells x (1-non-connected cells), where Total cells is the number of total cell in the field, connected cells is the number of the cells that form a tubular structures and non-connected cells is the number of the cells outside of tubular structures.

### Statistical analysis

Results are expressed as mean ± SEM. All values represent data from at least three independent cultures. Comparison between groups was performed using unpaired or paired Student *t-*test, one-way ANOVA followed by Bonferroni post-hoc test or two-ways ANOVA as appropriate. P < 0.05 was considered significant.

### Electronic supplementary material

Below is the link to the electronic supplementary material.

Additional File 1: Supplementary Figures

## Data Availability

The data that support the findings of this study are available from the corresponding author upon reasonable request.
