# Supplementary material for: Opening of Cx43-formed hemichannels mediates the Ca2+ signaling associated with endothelial cell migration
Source: Biol Direct. 2023 Aug 28;18:52. doi: 10.1186/s13062-023-00408-3 (PMC10463847; doi:10.1186/s13062-023-00408-3)
Supplement: Supplementary file 1 — Additional File 1: Supplementary Figures [file 13062_2023_408_MOESM1_ESM.pdf]

SUPPLEMENTARY FIGURES

Opening of Cx43-formed hemichannels mediates the Ca^{2+} signaling associated with endothelial cell migration

Hilda Espinoza[1,2] and Xavier F. Figueroa[1]*

[1]Departamento de Fisiología, Facultad de Ciencias Biológicas, Pontificia Universidad Católica de Chile, Santiago 8330025, Chile

[2]Escuela de Medicina, Facultad de Ciencias de la Salud, Universidad del Alba, Santiago 8370007, Chile

Running title: Cx43 hemichannel-mediated endothelial cell migration

***Author for correspondence:**

Dr. Xavier F. Figueroa

Departamento de Fisiología

Facultad de Ciencias Biológicas

Pontificia Universidad Católica de Chile

Santiago, Chile

Phone: 562-2686-2356

E-mail: xfigueroa@bio.puc.cl

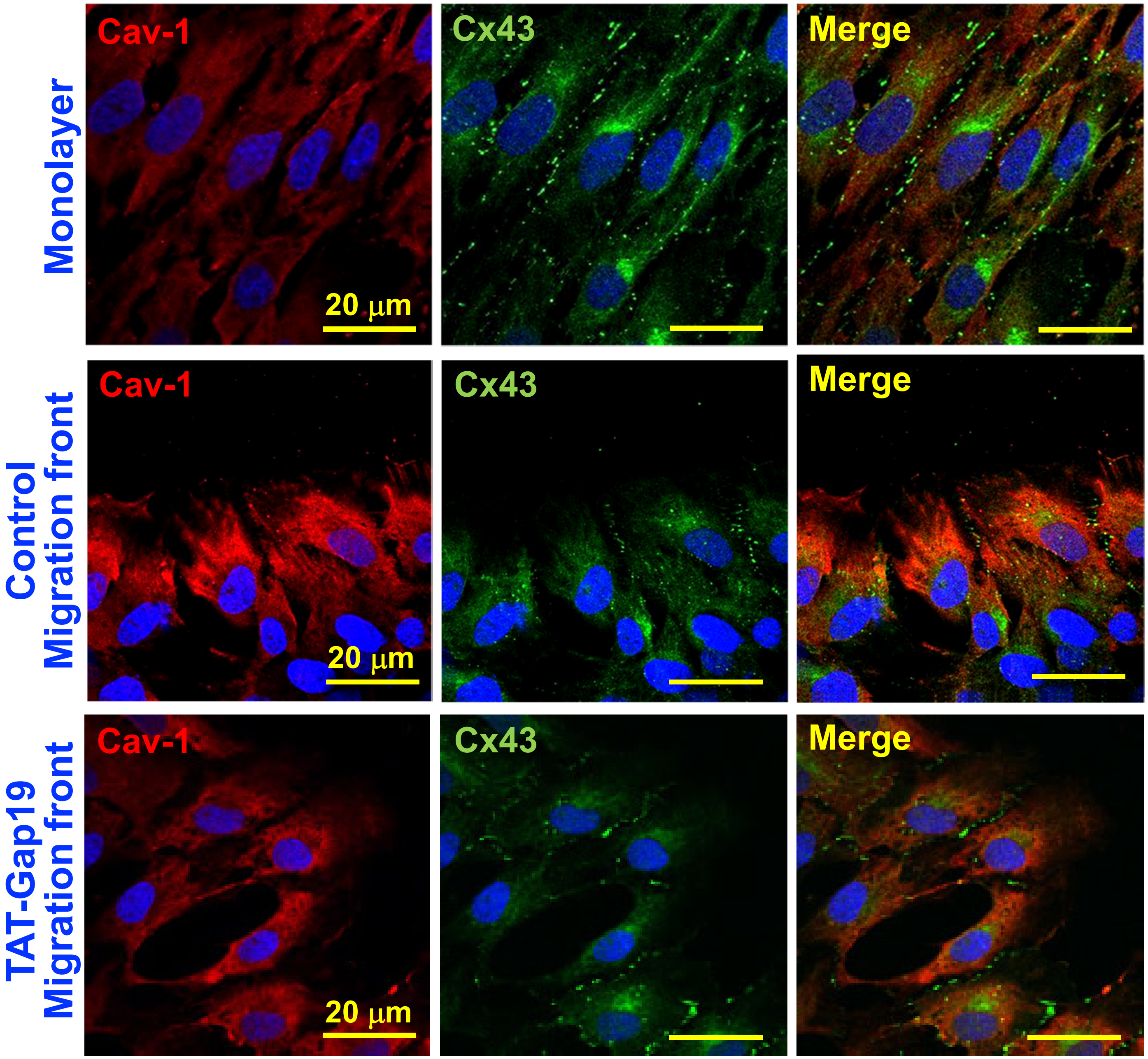

Supplementary Figure 1: Expression of Cx43 and caveolin-1 (Cav-1) in primary cultures of mesenteric endothelial cells. The subcellular distribution of Cx43 (green) and Cav-1 (red) was analyzed by immunofluorescence in the intact monolayer of endothelial cells and in the migration front 4 h after scratching the monolayer to initiate the wound-healing assay in control conditions or in the presence of 300 μM TAT-Gap19, a specific blocker of Cx43 hemichannels. Cell nuclei are highlighted by the staining with DAPI (blue).

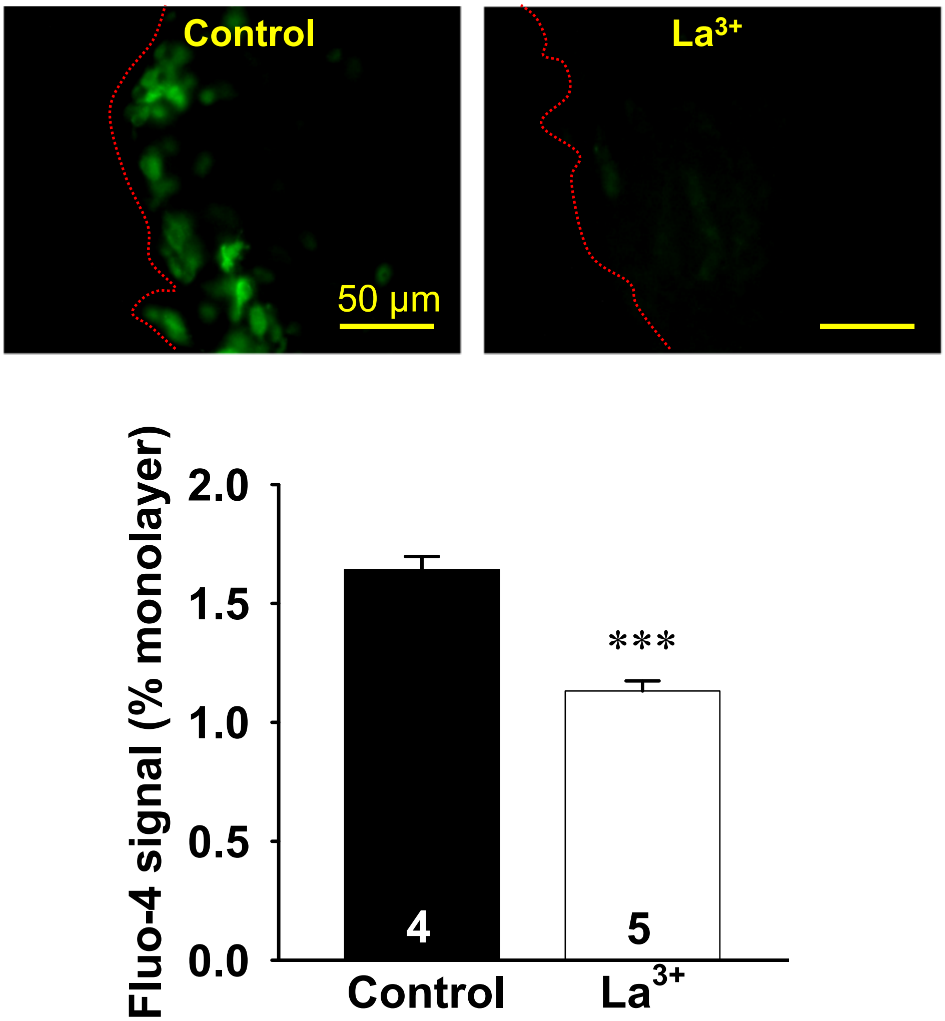

Supplementary Figure 2: The increase in intracellular Ca^{2+} concentration ($[Ca^{2+}]_i$) observed in endothelial cells of the migration front depends on the opening of Cx-formed hemichannels. Representative images (top) and fluorescence intensity analysis (bottom) of the increase in $[Ca^{2+}]_i$ observed in endothelial cells of the migration front 15 min after scratching the monolayer in control conditions and in the presence of 200 µM La^{3+}, a general blocker of Cx-formed hemichannels. Variations in the levels of $[Ca^{2+}]_i$ were assessed with the fluorescent Ca^{2+} indicator Fluo-4 and dot red lines depict the edge of the migration front. ***, $P < 0.001$ vs Control by unpaired Student's t-test

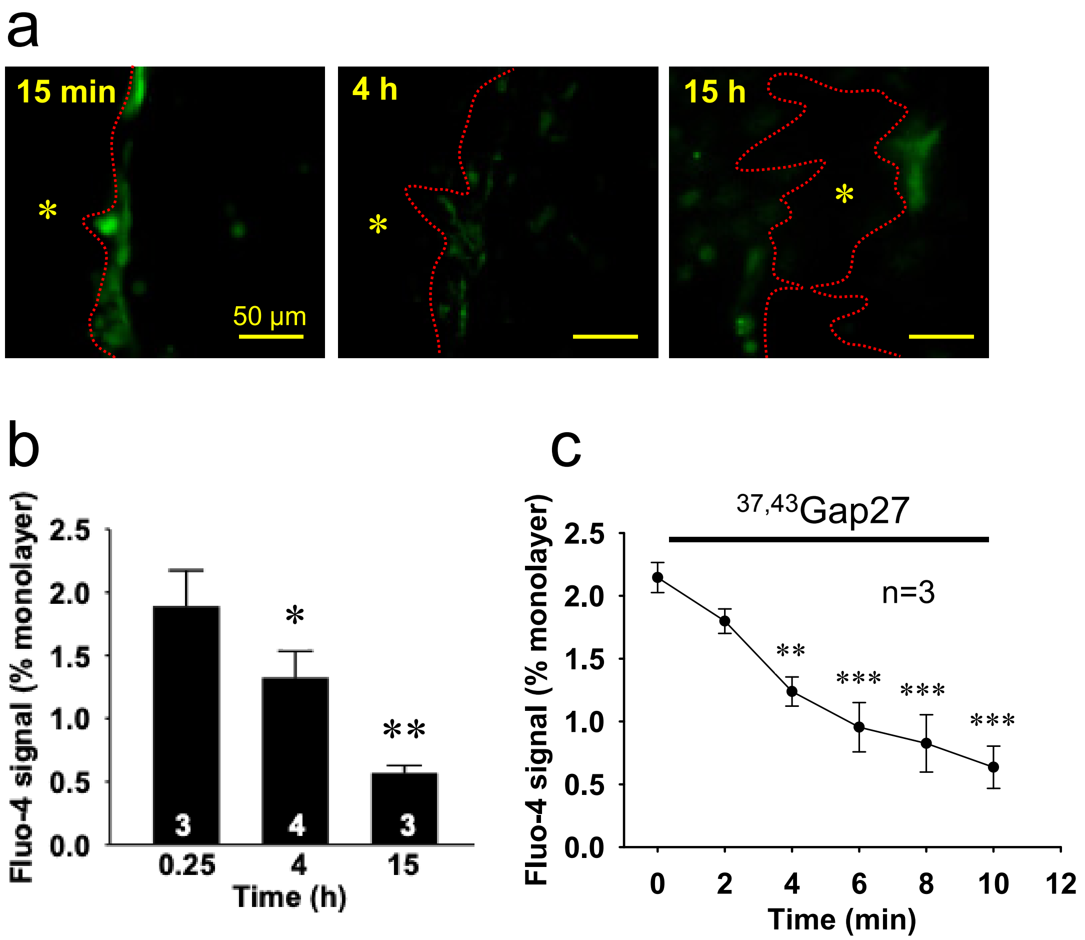

Supplementary Figure 3: Time course of Cx43 hemichannel-depended increase in intracellular Ca^{2+} concentration ($[Ca^{2+}]_i$) observed in endothelial cells of the migration front. **a and b**, Representative images (**a**) and fluorescence intensity analysis (**b**) of the increase in $[Ca^{2+}]_i$ observed in endothelial cells of the migration front 0.25 h (15 min), 4 h and 15 h after scratching the monolayer in control conditions. Yellow asterisks indicate the wounded area and dot red lines depict the edge of the migration front. Variations in the levels of $[Ca^{2+}]_i$ were assessed using the fluorescent Ca^{2+} indicator Fluo-4. In each time point, endothelial cells were uploaded with Fluo-4 through the incubation with 3 µM Fluo 4-AM for 1 h previous the experiment. **c**, Reduction of the increased $[Ca^{2+}]_i$ recorded along the time during the inhibition of Cx43-formed hemichannels by the treatment with 200 µM ^{37,43}Gap27. Note that application of the Cx blocking peptide ^{37,43}Gap27 evoked a rapid decay in the Fluo-4 fluorescence signal, which is consistent with the time course of the blockade of hemichannels, but not with the slow kinetic of the inhibition of gap junction channels (> 1 hr). *, $P < 0.05$; **, $P < 0.01$ and ***, $P < 0.001$, vs Control by one-way ANOVA plus Bonferroni post hoc test.

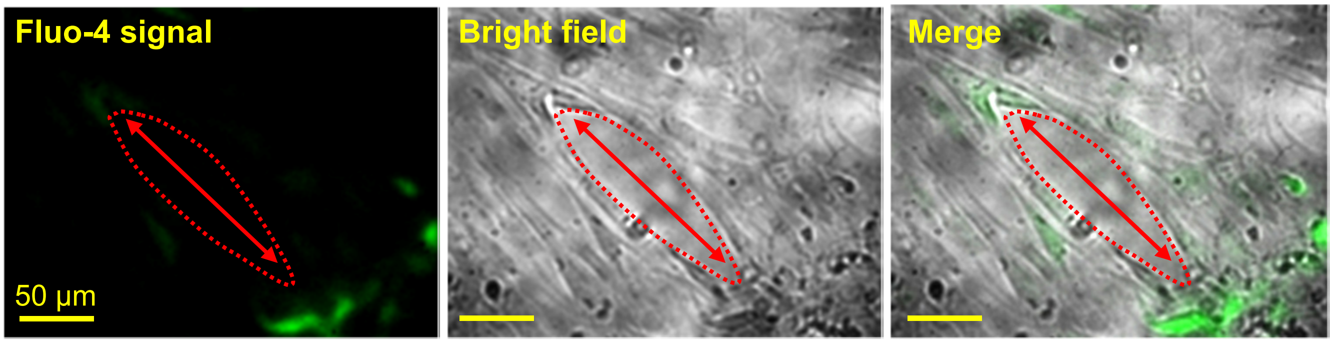

Supplementary Figure 4. The levels of intracellular Ca^{2+} concentration ($[Ca^{2+}]_i$) remain increased in endothelial cells of the migration front until the closure of the wounded area. The figure shows representative images of the increase in $[Ca^{2+}]_i$ observed in endothelial cells of the migration front 15 h after scratching the monolayer in control conditions. Variations in the levels of $[Ca^{2+}]_i$ were assessed using the fluorescent Ca^{2+} indicator Fluo-4 and dot red lines depict the edge of the migration front. Note that the wound is mostly closed and the increase in $[Ca^{2+}]_i$ is restricted to the cells found immediately around to the remaining open area (arrow).

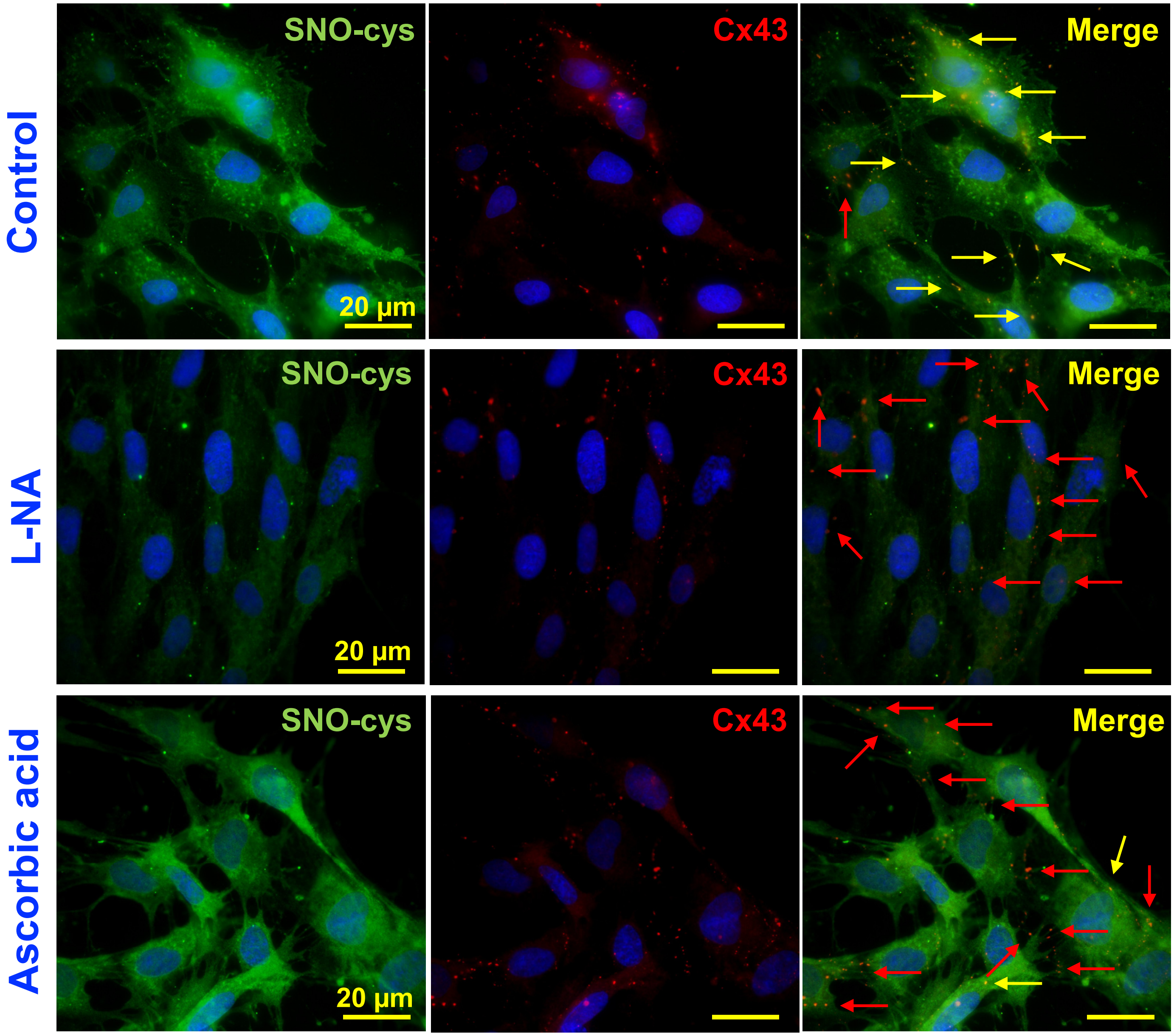

Supplementary Figure 5: Association of NO-mediated S-nitrosylation with Cx43 in endothelial cells of the migration front. The increase in total protein S-nitrosylation and the expression of Cx43 were detected by immunofluorescence in the migration front of primary cultures of endothelial cells 4 h after scratching the monolayer to initiate the wound-healing assay in control conditions or in the presence of 100 µM N^G-nitro-L-arginine (L-NA), a general blocker of NO production, or 50 µM ascorbic acid, a reducer that can denitrosylate proteins. Protein S-nitrosylation was detected using an antibody directed against SNO-cys (Sigma Aldrich, MO, USA). Yellow arrows highlight the co-localization of the signal for SNO-cys radicals and Cx43; in contrast, red arrows denote the presence of Cx43 signal alone. Cell nuclei are highlighted by the staining with DAPI (blue).
